# 2150. *In Vitro* Activity of Cefepime-Taniborbactam and Comparators Against Genotypically Characterized Carbapenem-Resistant Enterobacterales (CRE) and Carbapenem-Resistant *Pseudomonas aeruginosa* (CRPA) from the United States, 2018-2021

**DOI:** 10.1093/ofid/ofad500.1773

**Published:** 2023-11-27

**Authors:** Mark G Wise, Meredith Hackel, Daniel F Sahm

**Affiliations:** IHMA, Schaumburg, Illinois; IHMA, Schaumburg, Illinois; IHMA, Schaumburg, Illinois

## Abstract

**Background:**

Taniborbactam, a cyclic boronate-based β-lactamase inhibitor with activity against serine-, and NDM & VIM metallo-β-lactamases (MBLs), in combination with the fourth-generation cephalosporin, cefepime, is in development for treatment of complicated urinary tract infections. This study examined the *in vitro* activity of cefepime-taniborbactam against clinical isolates from the US, with a focus on carbapenem-resistant Enterobacterales (CRE) and carbapenem-resistant *Pseudomonas aeruginosa* (CRPA).

**Methods:**

From 2018-2021, as part of the GEARS Antimicrobial Surveillance Program, 4,220 Enterobacterales and 1,222 *P. aeruginosa* were collected from 38 clinical sites in the US. MICs of cefepime with taniborbactam fixed at 4 µg/mL and comparators were determined by broth microdilution according to CLSI guidelines and interpreted using 2023 CLSI breakpoints. CRE was defined by resistance to meropenem; CRPA was defined by resistance to meropenem and/or imipenem. Isolates with cefepime-taniborbactam MIC ≥16 µg/mL were characterized by whole genome sequencing. Isolates resistant to meropenem were screened for acquired β-lactamases by multiplex PCR/Sanger sequencing.

**Results:**

In total, 97.1% of the 68 CRE isolates were inhibited by ≤16 µg/mL of cefepime-taniborbactam. The majority of CRE (51/68; 75.0%) produced a carbapenemase (40 KPC, 9 MBLs, 2 OXA-48) and 98.0% were inhibited by ≤16 µg/mL cefepime-taniborbactam, a substantially greater percentage than the most active comparator, meropenem-vaborbactam (82.4% susceptible). Cefepime-taniborbactam at ≤16 µg/mL inhibited 92.9% of all CRPA (n=308), 88.7% of meropenem-resistant *P. aeruginosa* (n=177), and 90.6% of carbapenemase-negative, meropenem-resistant *P. aeruginosa* (n=170) whereas the most active comparator, ceftazidime-avibactam, covered 84.7%, 75.7%, and 77.6% of these resistant subsets, respectively.

**Figure:**
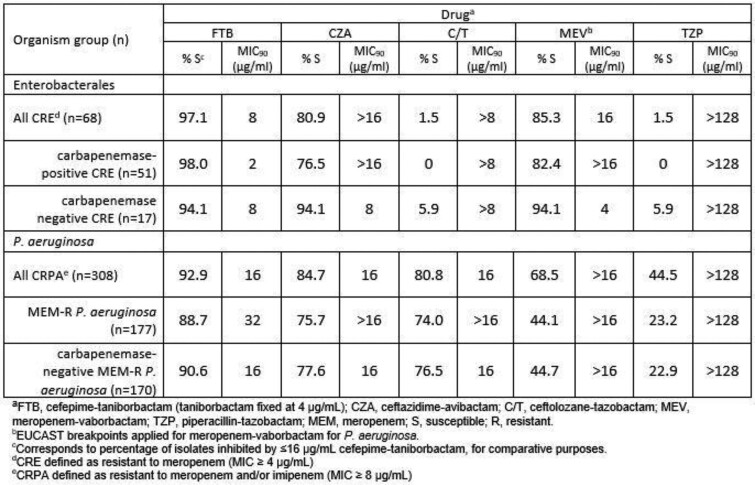

**Conclusion:**

Cefepime-taniborbactam inhibited ≥94.1% of CRE isolates from the US, regardless of carbapenemase carriage. Similarly potent activity was observed for cefepime-taniborbactam against CRPA, including meropenem-resistant strains without a detected carbapenemase. The continued development of cefepime-taniborbactam appears warranted.

**Disclosures:**

**Mark G Wise, PhD**, Merck & Co., Inc.: Honoraria|Pfizer Inc.: Honoraria|Venatorx: Paid fees for conducting the study and abstract preparation **Meredith Hackel, PhD**, Pfizer Inc.: Honoraria|Venatorx: Paid fees for conducting the study and abstract preparation **Daniel F. Sahm, PhD**, Merck & Co., Inc.: Honoraria|Pfizer Inc.: Honoraria|Venatorx: Paid fees for conducting the study and abstract preparation

